# Supplementary figures and images for: Design of a Tension Infiltrometer with Automated Data Collection Using a Supervisory Control and Data Acquisition System
Source: Sensors (Basel). 2023 Nov 29;23(23):9489. doi: 10.3390/s23239489 (PMC10708719; doi:10.3390/s23239489)

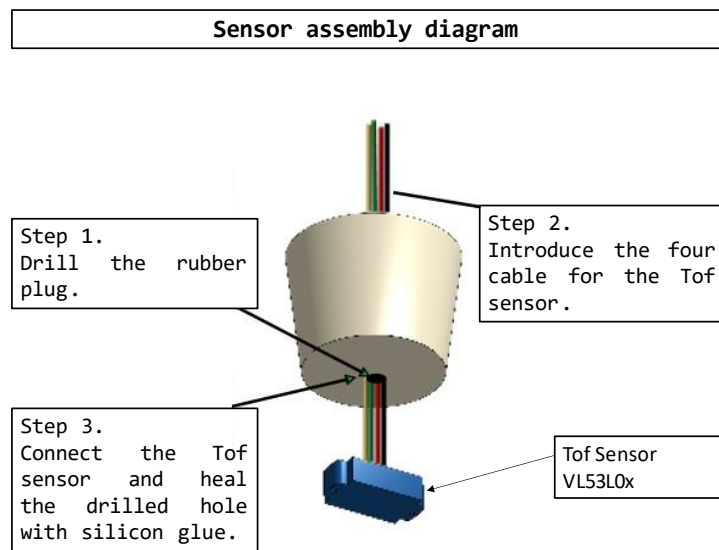

**Figure S1.** Sensor assembly.

Supplement: Supplementary file 1 [file sensors-23-09489-s001.zip › sensors-2585418-supplementary.pdf]
